# Analysis of nocturnal desaturation waveforms using algorithms in patients with idiopathic pulmonary fibrosis

**DOI:** 10.1007/s11325-021-02456-3

**Published:** 2021-08-21

**Authors:** Yuichiro Yasuda, Tatsuya Nagano, Shintaro Izumi, Mina Yasuda, Kosuke Tsuruno, Kazunori Tobino, Kyosuke Nakata, Kayoko Okamura, Teruaki Nishiuma, Kiyonobu Takatsuki, Yasuhiro Funada, Hisashi Ohnishi, Masatsugu Yamamoto, Yoshihiro Nishimura, Kazuyuki Kobayashi

**Affiliations:** 1 Department of Internal Medicine, Division of Respiratory Medicine, Kobe University Graduate School of Medicine, 7-5-1 Kusunoki-cho, Chuo-ku, Kobe, Hyogo 650-0017 Japan; 2 Graduate School of System Informatics, Kobe University, 1-1-Rokkodai-cho, Nada-ku, Kobe, Hyogo 657-8501 Japan; 3 Department of Respiratory Medicine, Iizuka Hospital, 3-83 Yoshiomachi, Iizuka, Fukuoka 820-0018 Japan; 4 Department of Respiratory Medicine, Kasai City Hospital, 1-13 Yoko, Hojo-cho, Kasai, Hyogo 675-2311 Japan; 5 Department of Respiratory Medicine, Akashi Medical Center, 743-33 Yagi, Okubo-cho, Akashi, Hyogo 674-0063 Japan; 6 Department of Respiratory Medicine, Kakogawa Central City Hospital, 439 Honmachi, Kakogawa-cho, Kakogawa, Hyogo 675-8611 Japan; 7 Department of Respiratory Medicine, Kitaharima Medical Center, 926-250 Ichiba-cho, Ono, Hyogo 675-1392 Japan; 8 Department of Respiratory Disease, Takatsuki General Hospital, 1-3-13 Kosobe-cho, Takatsuki, Osaka 569-1192 Japan

**Keywords:** Home sleep apnea test, Idiopathic pulmonary fibrosis, Nocturnal hypoxemia, Percutaneous oxygen saturation

## Abstract

**Purpose:**

Sleep-disordered breathing is recognized as a comorbidity in patients with idiopathic pulmonary fibrosis (IPF). Among them, nocturnal hypoxemia has been reported to be associated with poor prognosis and disease progression. We developed a diagnostic algorithm to classify nocturnal desaturation from percutaneous oxygen saturation (SpO_2_) waveform patterns: sustained pattern, periodic pattern, and intermittent pattern. We then investigated the prevalence of nocturnal desaturation and the association between the waveform patterns of nocturnal desaturation and clinical findings of patients with IPF.

**Methods:**

We prospectively enrolled patients with IPF from seven general hospitals between April 2017 and March 2020 and measured nocturnal SpO_2_ and nasal airflow by using a home sleep apnea test. An algorithm was used to classify the types of nocturnal desaturation. We evaluated the association between sleep or clinical parameters and each waveform pattern of nocturnal desaturation.

**Results:**

Among 60 patients (47 men) who met the eligibility criteria, there were 3 cases with the sustained pattern, 49 cases with the periodic pattern, and 41 cases with the intermittent pattern. Lowest SpO_2_ during sleep and total sleep time spent with SpO_2_ < 90% were associated with the sustained pattern, and apnea–hypopnea index was associated with the intermittent pattern.

**Conclusion:**

We demonstrated the prevalence of each waveform and association between each waveform and sleep parameters in patients with IPF. This classification algorithm may be useful to predict the degree of hypoxemia or the complication of obstructive sleep apnea.

**Supplementary Information:**

The online version contains supplementary material available at 10.1007/s11325-021-02456-3.

## Introduction

Idiopathic pulmonary fibrosis (IPF) is defined as a specific form of chronic, progressive, fibrosing interstitial pneumonia of unknown cause [1]. The median survival time after diagnosis has been reported to be approximately 35 months [2]. The management of various comorbidities with IPF is a clinically important issue. Obstructive sleep apnea (OSA) is one of the comorbidities [3]. However, less research has been focused on changes in oxygenation and breathing patterns in IPF during sleep compared to daytime oxygenation. Including OSA, nocturnal hypoxemia is reported to be observed occasionally in IPF patients [4]. Troy and colleagues showed that nocturnal hypoxemia is associated with poor prognosis and may contribute to the development of pulmonary hypertension in interstitial lung disease (ILD) patients [5]. Also, the presence of OSA with nocturnal hypoxemia has been reportedly associated with poor prognosis and disease progression in IPF patients [6]. Pulmonary function tended to be worse in the group with both OSA and nocturnal hypoxemia than in the group without sleep breathing disorder and the group with OSA only. These observations suggest the association between progression in nocturnal disorder and the disease stage of IPF. The recognition of nocturnal hypoxemia in IPF patients might shed light not only on diagnosing such complications but also on associating the disease progression itself of IPF.

**Fig. 1:**
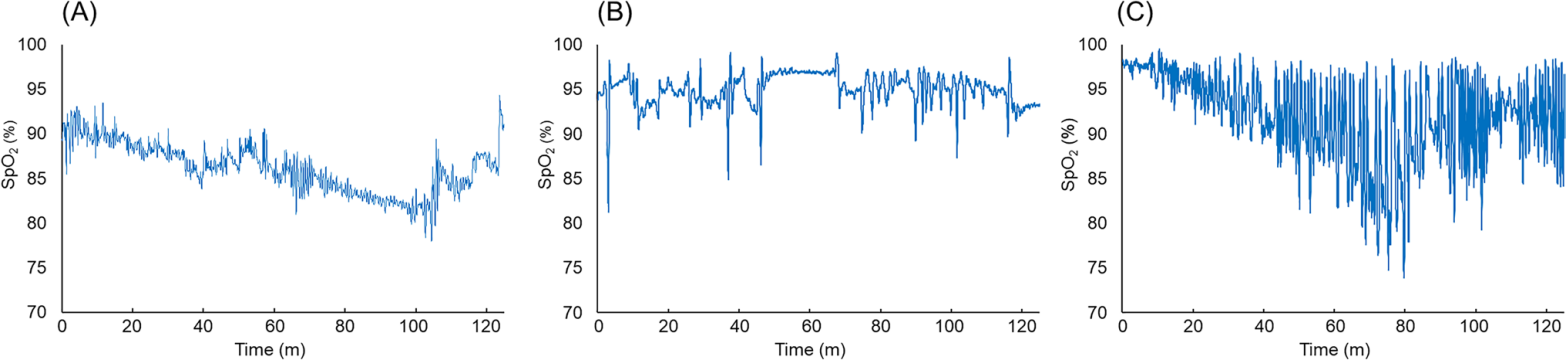
Examples of waveforms of nocturnal desaturation. Sustained pattern (**A**), periodic pattern (**B**), and intermittent pattern (**C**)

In our previous study, we monitored nocturnal percutaneous oxygen saturation (SpO_2_) level in the patients with chronic respiratory disease that needed home oxygen therapy (HOT) [7]. This study confirmed that nocturnal desaturation occurred even in the patients in whom HOT improved daytime hypoxemia. Also, the waveform pattern differed greatly among SpO_2_ waveform patterns. Then, we developed a diagnostic algorithm according to the pathophysiological condition for SpO_2_ waveform patterns: sustained pattern, periodic pattern, and intermittent pattern (examples of the waveforms are presented in Fig. 1) [7], on the basis of the respiratory pathophysiology under HOT, including the duration of desaturation and frequent repetition of drop and recover in SpO_2_. Although the algorithm was developed based on the oxygen therapy, we sought that the pathophysiology on the desaturation fluctuation could be applied to other respiratory diseases even without oxygen therapy. Since it has not yet been investigated in individual respiratory diseases without oxygen therapy, this study focused on IPF. We aimed to investigate the prevalence of nocturnal desaturation in IPF and to determine the association between the waveform patterns of nocturnal desaturation and clinical findings.

## Patients and methods

### Patients

**Fig. 2:**
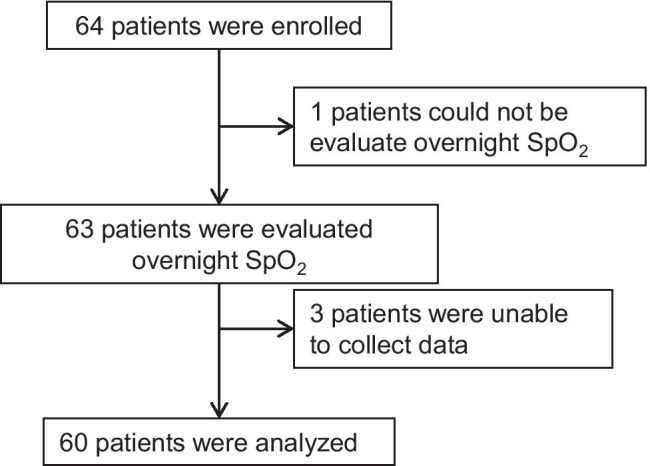
Flow chart of patient recruitment process

This multicenter prospective study was performed between April 2017 and March 2020 at seven general hospitals in Japan, namely, Kobe University Hospital, Iizuka Hospital, Kasai City Hospital, Kakogawa Central City Hospital, Kitaharima Medical Center, Akashi Medical Center, and Takatsuki General Hospital. We enrolled 64 outpatients with IPF who met the following criteria: were aged 20 years or older; were not undergoing HOT; had daytime SpO_2_ > 88% or partial pressure of oxygen in arterial blood (PaO_2_) > 55 torr. IPF was diagnosed based on international guidelines [1]. A flowchart showing the patient recruitment process is shown in Fig. 2. This study was approved by the institutional review board at Kobe University Hospital (approval number: 170017).

### Home sleep apnea test

SpO_2_ and nasal airflow were recorded continuously using a SAS-2100 device (Nihon Kohden Corporation, Tokyo, Japan). Apnea–hypopnea index (AHI, average apnea and hypopnea per hour of sleep time), total sleep time, and lowest SpO_2_ were recorded. Apnea is defined as a reduction in signal amplitude to 10% of baseline for at least 10 s. Hypopnea is defined as a reduction in signal amplitude to 70% of baseline and ≥ 3% oxygen desaturation for at least 10 s. Patients with a total sleep time of less than 4 h were excluded from the analysis.

### Pulmonary function test

Pulmonary function test (PFT) and the measurement of diffusing capacity for carbon monoxide (DLCO) were performed according to the international recommendations [8, 9] within 3 months of enrollment in each hospital. Japanese local reference values were used for the predicted values of PFT [10].

### Six-minute walk test

The six-minute walk test (6MWT) was performed in all patients by trained technicians. The 6-min walk distance and lowest SpO_2_ value were recorded.

### Other clinical data

Gender, height, weight, comorbidities, smoking history, medications, daytime SpO_2_ and PaO_2_ at rest, echocardiography-measured ejection fraction and transtricuspid pressure gradient (TRPG), subjective breathlessness scale (modified medical research council), and laboratory data including sialylated carbohydrate antigen Krebs von den Lungen-6 and surfactant protein-D were measured or extracted from patients’ medical record.

### Severity of IPF

IPF patients were classified as stage I (PaO_2_ > 80 mmHg at rest), stage II (70–79 mmHg), stage III (60–69 mmHg), or stage IV (< 59 mmHg) based on the severity stage used clinically in Japan. Among patients with stage II or III, the severity is increased by one stage if the lowest SpO_2_ is less than 90% during a 6MWT [11].

### Classification algorithm of nocturnal desaturation

**Fig. 3:**
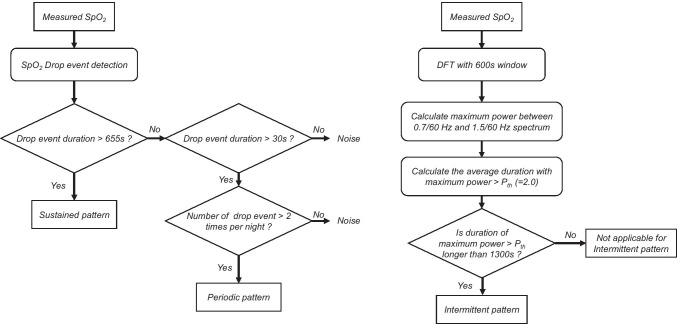
Flowchart of nocturnal desaturation pattern judgment

Desaturation was defined as the decrease in SpO_2_ by more than 3% from baseline. The cases with nocturnal desaturation were diagnosed based on the reported algorithm into three classifications, namely, sustained pattern, periodic pattern, and intermittent pattern [7]. Briefly, when one or more drop events longer than 655 s occur, the measured data were labeled as the sustained pattern. When the drop event between 30 and 655 s occurred more than twice during the measurement period, the pattern was labeled as the periodic pattern. We defined the third pattern for characteristic frequency pattern seen in OSA, intermittent pattern where the drop and recovery in SpO_2_ was repeated with a cycle of several minutes. In addition, the time–frequency analysis using discrete Fourier transform was conducted for SpO_2_ data (Figure S1). The window length was set to 600 s and the maximum spectral power between 0.7/60 and 1.5/60 Hz is calculated. The intermittent pattern was set if the maximum spectral power was larger than 2.0 and this state occurred for more than 1300 s per hour. Each pattern of desaturation can overlap in a patient because of the distinct algorithm which is not exclusive of the other patterns. The flowchart of the classification for nocturnal desaturation is shown in Fig. 3.

### Sample size

According to previous reports, 37% of patients with ILD showed nocturnal hypoxemia [12]. The proportions of cases assigned to stages I, II, III, and IV were 43%, 13%, 22%, and 22%, respectively [13]. Here, we hypothesized that the prevalence of nocturnal desaturation depends on the IPF severity. Since there was a difference in the prognosis between the cases in stages I/II and III/IV, we divided the current patients into two groups. In our hypothesis, we estimated that the prevalence rates of nocturnal hypoxemia in stage I/II and stage III/IV are 20% and 60%, respectively. We estimated that 31 and 24 patients were needed in the groups with a two-sided alpha value of 0.05 and a statistical power value of 80%.

### Statistical analysis

Fisher’s exact test was used for between-group comparisons of the categorical variables. As exploratory analyses, logistic regression analysis and multiple regression analysis were conducted to determine clinical parameters associated with each waveform classification. All *P* values reported are 2-sided, and *P* values less than 0.05 were considered significant. All of the statistical analyses were performed with EZR (Saitama Medical Center, Jichi Medical University), which is a graphical user interface for the R software program (The R Foundation for Statistical Computing, version 2.13.0) [14]. It is a modified version of R commander (version 1.27) that includes statistical functions that are used in biostatistics.

## Results

### Patient characteristic

Of 60 patients (47 men) who met the eligibility criteria (Table 1), the number of patients with stages I/II and III/IV was 43 (72%) and 17 (28%), respectively. The mean forced vital capacity (FVC) and DLCO were 81% and 61% predicted, respectively. Thirty-two patients underwent TTE, but no patients had elevated TRPG; therefore, we did not analyze the association of pulmonary hypertension in this study.

**Table 1:** Patients’ characteristics (*n* = 60)

Sex	
Male/female	47/13
Age, yr	72.5 ± 8.6
BMI	24.1 ± 3.2
Smoking status, *n* (%)	
Never/former/current	16 (26.7)/39 (65)/5 (8.3)
Daytime PaO_2_ (mmHg)	83.9 ± 11.9
Daytime SpO_2_ (%)	96.5 ± 1.4
IPF severity, *n* (%)	
I/II/III/IV	36 (60)/7 (11.7)/12 (20)/5 (8.3)
mMRC, *n* (%)	
0/1/2/3/4	16 (26.7)/21 (35)/13 (21.7)/10 (16.7)/0 (0)
Pulmonary function test	
VC, % predicted	79.5 ± 17.3
FVC, % predicted	81 ± 17.7
FEV1, % predicted	84 ± 19.4
DLCO, % predicted	60.8 ± 16.9
6MWT	
Distance (m)	372 ± 120
Lowest SpO_2_ (%)	90 ± 4.5
KL-6 (U/ml)	937 ± 673
SP-D (ng/ml)	223 ± 147
TRPG > 40 mmHg on TTE, n/total	0/32
Past medical history, n (%)	
Hypertension	28 (46.7)
Diabetes mellitus	19 (31.7)
Gastroesophageal reflux disease	1 (1.6)
Ischemic heart disease	5 (8.3)
Chronic obstructive pulmonary disease	4 (6.7)
Lung cancer	5 (8.3)
Depression	0 (0)
Neuromuscular disorder	0 (0)
Medication, *n* (%)	
Oral corticosteroids	3 (5)
Sleeping medicine	2 (3.3)
Anti-fibrotic drug	23 (38.3)

The data are expressed as the number or mean ± SD

*BMI*, body mass index, *IPF*, idiopathic pulmonary fibrosis, *VC*, vital capacity, *FVC*, forced vital capacity, *FEV1*, forced expiratory volume in one second, *DLCO*: diffusing capacity for carbon monoxide, *6MWT*, six-minute walk test, *TRPG*, transtricuspid pressure gradient, *TTE*, transthoracic echocardiography

### Nocturnal desaturation and its classification

**Fig. 4:**
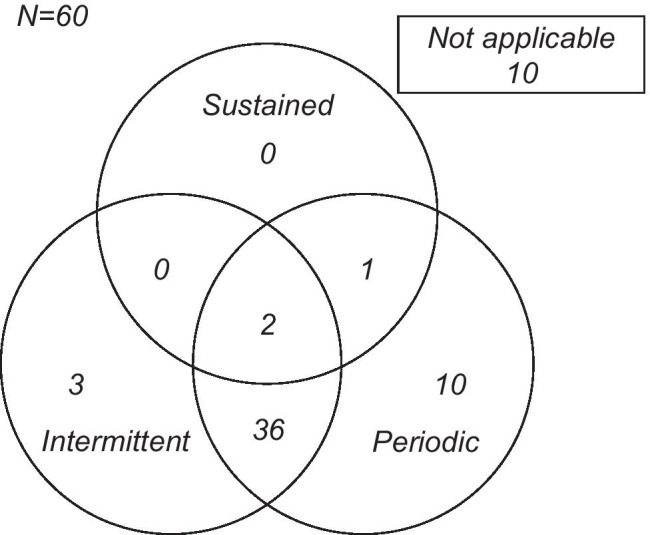
Venn diagram of each waveform in patients with idiopathic pulmonary fibrosis

Of 60 cases, 52 (87%) corresponded with any of the nocturnal desaturation patterns. The sleep characteristics are summarized in Table 2. The number of patients showing nocturnal desaturation was 3 (5%), 49 (82%), and 41 (68%) with sustained pattern, periodic pattern, and intermittent pattern, respectively. There were eight patients who did not meet each pattern criteria. A Venn diagram for the waveform pattern is shown in Fig. 4. For the IPF severity, in severity stage I/II (43 patients), there were 2 patients (4%), 35 patients (81%), and 29 patients (67%), in the sustained, periodic, and intermittent patterns, respectively. Also, in severity stage III/IV (17 patients), there were 1 patient (6%), 14 patients (82%), and 12 patients (71%), respectively. There was no significant difference between stages I/II and III/IV regarding the waveform patterns. The clinical and sleep characteristics with sustained pattern are described in Table S1. Only 3 patients showed desaturation with the sustained pattern. The DLCO% predicted decreased in all three cases. Moreover, one of the three patients showed an AHI of 6.3. Separately, we analyzed 10 healthy subjects using the algorithm for the waveform classifications. All healthy subjects met none of the classifications for nocturnal desaturation (Table S2).

**Table 2:** Sleep characteristics

Total sleep time (min)	455 ± 90
AHI	19.3 ± 13.8
Lowest SpO_2_ (%)	81.5 ± 6.5
TST90 (%)	1.3 (0.1–3.1)
Classification of nocturnal desaturation, *n* (%)	
Sustained	3 (5)
Periodic	49 (82)
Intermittent	41 (68)
Not applicable	8 (13)

The data are expressed as the number or mean ± SD or median (interquartile range)

*AHI*, apnea–hypopnea index, *TST90*, total sleep time with SpO_2_ < 90%

### Association between nocturnal oxygen saturation and the waveform patterns

As exploratory analyses to assess the association between the classifications by the algorithm and nocturnal oxygen saturation, we performed the logistic regression analyses for modeling each waveform pattern. AHI, lowest SpO_2_, and TST90 were included as predictor variables. Lowest SpO_2_ and TST90 were associated with the sustained pattern (lowest SpO_2_, odds ratio (OR): 0.796, 95% confidence interval (CI): 0.637–0.994, *P* value = 0.04; TST90, OR: 1.1, 95% CI: 1.01–1.18, *P* value = 0.02). AHI was associated with the intermittent pattern (AHI, OR: 1.54, 95% CI: 1.16–2.06, *P* value < 0.005). They were not significantly associated with the periodic pattern.

### Association between daytime clinical parameters and the waveform patterns

Next, in order to analyze the association between the daytime clinical parameters and each waveform pattern, we conducted the logistic regression analysis and multivariable regression analysis. No significant associations were found between lower DLCO (< 60% predicted) and any of the waveform patterns. FVC (% predicted) tended to be higher, although not significantly, in the intermittent group compared with the others (*P* value = 0.07).

## Discussion

In the present study, we prospectively analyzed the waveform patterns in nocturnal desaturation in patients with IPF. There was no significant difference between stages I/II and III/IV regarding the waveform patterns. Persistent nocturnal hypoxemia was less than a priori estimation, as the persistent hypoxemia was observed less in the IPF patients without HOT than in the patients with HOT as reported [7]. Focusing on the SpO_2_ fluctuation at night, however, we did observe nocturnal desaturation events in most of the IPF patients. These results suggest a subclinical disordered respiration even in IPF patients without hypoxemia.

Exploratory analysis of waveform classification and sleep parameters suggested that the degree and duration of nocturnal desaturation (lowest SpO_2_ and TST90) was associated with the sustained pattern. Three patients with the sustained pattern had decreased DLCO; however, preserved AHI despite sustained desaturation in one of such patients might shed light on the difficulty in assessing nocturnal hypoxemia solely by AHI. A recent study on nocturnal hypoxemia in ILD reported that TST90 correlated significantly with several markers of ILD disease severity including oxygenation and DLCO [5]. In addition, it may be necessary to verify whether the waveform pattern changes when continuous oxygen therapy is given to patients with sustained pattern.

Together with the algorithm setting that the sustained pattern is defined as desaturation over 10 min, these findings raise speculation that the sustained pattern might appear in IPF patients with relatively progressive disease, but sample size limited the further analysis in the current study. Future studies to optimize the algorithm including cut-off value of duration might lead to clarify the pathophysiological link to the sustained pattern and disease progression.

Our results showing that the intermittent pattern was associated with AHI suggested that this classification may predict the degree of hypoxemia or the complication of OSA. None of the sleep parameters showed a significant association with the periodic pattern.

Since the intermittent pattern correlates with AHI, this pattern seems to reflect sleep apnea. In the present study, intermittent pattern was found in 68%, which is considered to be a similar prevalence of OSA as reported previously [6, 15]. Given that it is necessary to recognize OSA in IPF patients, nocturnal desaturation with intermittent pattern might predict OSA or related disorder. In patients with the intermittent pattern, FVC% predicted tended to be maintained compared to the other classifications. It suggests that the waveform can change from an intermittent to a sustained pattern as the clinical course of IPF progression. The follow-up observational survey tracing nocturnal desaturation pattern might reveal the natural history of sleep-related disorder in IPF.

The periodic patterns were primarily assumed in the development as the desaturation events including rapid eye movement sleep-related hypoventilation. Primary observation revealed that many patients with HOT have periodic pattern [7]. In the current study, the periodic pattern was neither specific to IPF patients nor related to daytime and sleep parameters. Hence, the clinical implication of this waveform pattern was not clear in this study. Further study using polysomnography (PSG) for waveform classification may help elucidate the pathophysiology of periodic pattern.

The SAS-2100 is a portable sleep apnea tester which is capable of measuring airflow, SpO_2_, and pulse rate. The total sleep time is determined from the time the machine is turned on to off. In addition, this SAS-2100 cannot detect the degree of ventilation and the level of carbon dioxide. Therefore, the limitations of the present study are that the present study lacks data on PSG or overnight transcutaneous carbon dioxide tension. Home sleep apnea test tends to underestimate AHI because the recording time is longer than sleep time. There is also the inaccuracy that hypopnea associated with arousal is measured. Regarding waveform classification, whose cut-off value was set from the examination of HOT patients, it has not been verified for application in patients with IPF.

## Conclusions

We observed the prevalence of nocturnal desaturation and its classification in patients with IPF. The proof-of-concept study for the waveform algorithm showed that the patterns were related to the degree of hypoxemia or the complication of OSA, which can affect the prognosis in IPF. Future studies, including therapeutic interventions or PSG assessments, may reveal clinical significance of waveform classification.

## Supplementary Information

Below is the link to the electronic supplementary material.

Supplementary file1 Spectrogram of SpO2 waveform. Frequency analysis was performed using the Discrete Fourier Transform. Brighter background colors indicate the stronger power of the frequency component. The red line shows the peak frequency at each time (PPTX 220 KB)

Supplementary file2 (XLSX 12 KB)

Supplementary file3 (XLSX 11 KB)

## Data Availability

Data are available from the corresponding author upon reasonable request.

## Abbreviations

6MWT: The six-minute walk test
AHI: Apnea–hypopnea index
DLCO: Diffusing capacity for carbon monoxide
FVC: Forced vital capacity
HOT: Home oxygen therapy
ILD: Interstitial lung disease
IPF: Idiopathic pulmonary fibrosis
OSA: Obstructive sleep apnea
PaO_2_: Partial pressure of oxygen in arterial blood
PFT: Pulmonary function test
PSG: Polysomnography
SpO_2_: Percutaneous oxygen saturation
TRPG: Transtricuspid pressure gradient
TST90: Total sleep time spent with SpO_2_ < 90
