# Supplementary figures and images for: Analysis of nocturnal desaturation waveforms using algorithms in patients with idiopathic pulmonary fibrosis
Source: Sleep Breath. 2021 Aug 21;26(3):1079–86. doi: 10.1007/s11325-021-02456-3 (PMC9418279; doi:10.1007/s11325-021-02456-3)

## Slide 1
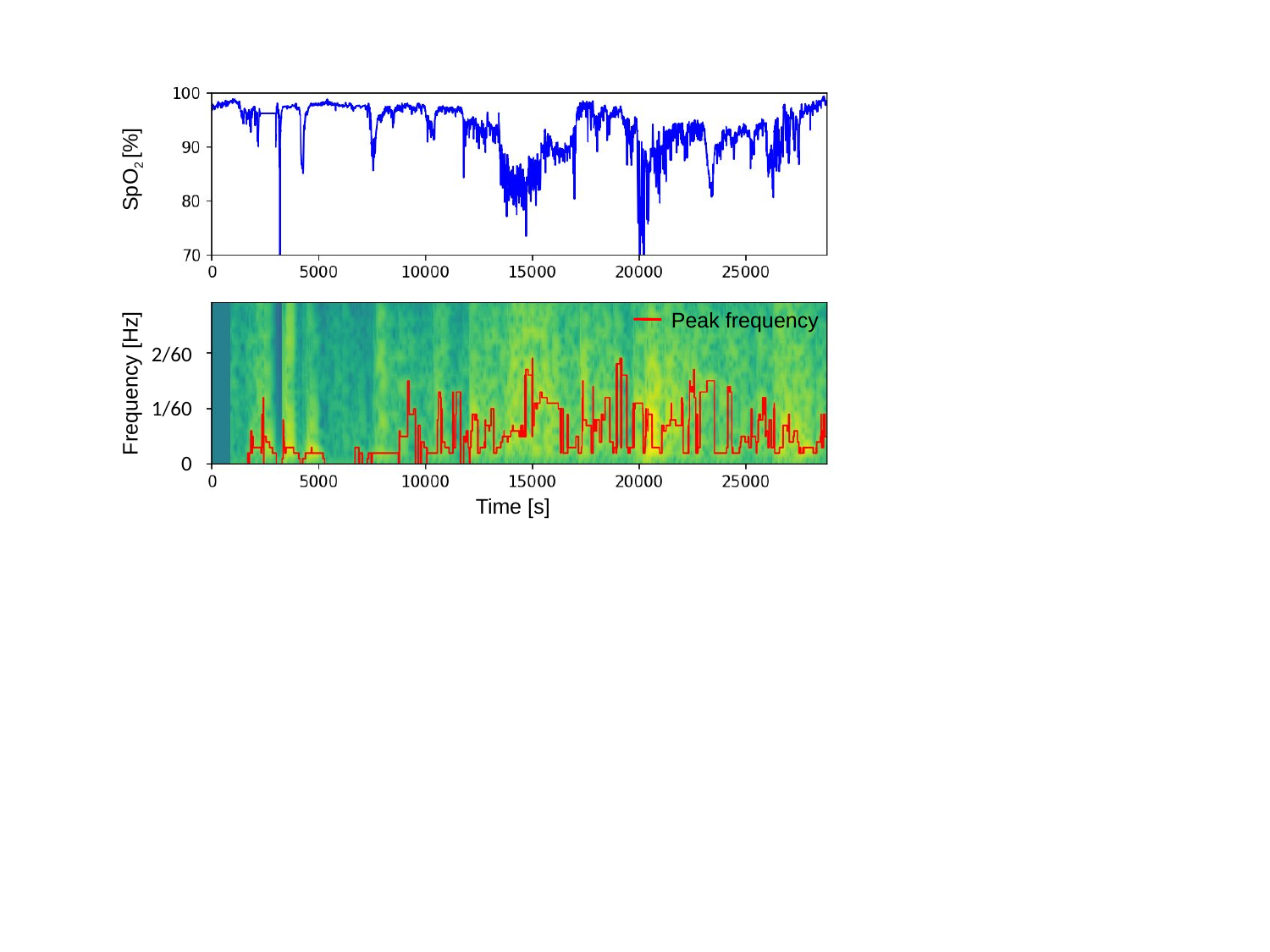

SpO2 [%]
Peak frequency
2/60
Frequency [Hz]
1/60
0
Time [s]

Supplement: Supplementary file 1 — Supplementary file1 Spectrogram of SpO2 waveform. Frequency analysis was performed using the Discrete Fourier Transform. Brighter background colors indicate the stronger power of the frequency component. The red line shows the peak frequency at each time (PPTX 220 KB) [file 11325_2021_2456_MOESM1_ESM.pptx]
